# A Pneumonia Creed

**Published:** 1918-11

**Authors:** 


### A PNEUMONIA CREED

A medical creed adapted to present conditions in France has been sent out from the Chief Surgeon's Office. It presents in concise form a code of action which, if lived up to by every medical officer of the American Expeditionary Forces, will reduce influenza and pneumonia rates enormously, thus saving the lives of many good Americans who need and deserve protection in this serious epidemic of influenza which is sweeping over Europe.

This Creed, extracted from the Weekly Bulletin on Disease of October 7th, is published below, and physicians who are charged with caring for the health and lives of our fighting forces should heed its teachings.

### A Medical Creed.

"Please learn it and hide not the light of your wisdom under a bushel.

Teach what you know, by personal example, and by word of mouth. Don't be afraid of boring your colleagues in the officers' mess. Be a fanatic on the subject of grippe, pneumonia and meningitis from now until next May. By becoming a nuisance in the command, you may easily be voted the best friend of every soldier.

*Notes for a daily lecture to some group of officers or men by every medical officer in the American Expeditionary Forces.*

1. Pneumonia is now causing more deaths than all other diseases in the American Expeditionary Forces put together. Together with influenza, colds and meningitis, it causes more days' loss than all other diseases and injuries combined. Diseases always cause more loss of life and days' service than do battle casualties in every army.

2. Pneumonia is preventable, because it is spread from sick to well just the way we catch colds, by breathing in each other's infected mouth and nose spray.

3. Pneumonia is preventable, because among men who are well-cared for the natural resistance against the infection is high.

4. There are always cases of respiratory infections in every body of men, however carefully selected.

5. The maintenance of the natural and remarkable effective bodily resistance to pneumonia and meningitis at the highest point, and the instant segregation of every case of cough and cold can at least be undertaken with confidence.

6. Tired, driven, anxious men; cold, wet, hungry men; crowded, smelly, huddled men; sneezing, coughing, spitting men get, and give pneumonia easily.

Let not mere circumstances determine the sick rate among your men. Only a morbid craving for notoriety is satisfied by reporting the highest mortality from pneumonia in the American Expeditionary Forces. Let your report show results from prevention, rather than the confession of helplessness in the face of a serious and immediately pressing danger."

